# Takotsubo Cardiomyopathy Associated with Polycythemia Vera

**DOI:** 10.1155/2018/4542925

**Published:** 2018-09-20

**Authors:** Sijan Basnet, Priya Rajagopalan, Rashmi Dhital, Biswaraj Tharu

**Affiliations:** ^1^Department of Medicine, Reading Hospital, 420 S Fifth Avenue, West Reading, PA 19611, USA; ^2^Jefferson Medical College, 1025 Walnut Street, Philadelphia, PA 19107, USA; ^3^Maharajgunj Medical Campus, Tribhuvan University, Kathmandu, Nepal

## Abstract

Takotsubo cardiomyopathy is characterized by transient apical ballooning with preserved basal ventricular function triggered by physical or emotional stressors. We present a case of a 75-year-old man referred to our facility for the management of acute myocardial infarction later diagnosed as takotsubo cardiomyopathy. We believe platelet-mediated adrenaline release from massive thrombocytosis might have been the precipitating factor for the pathogenesis of takotsubo cardiomyopathy.

## 1. Introduction

Takotsubo cardiomyopathy (TTC) was first reported by Sato et al. in 1990 [1]. TTC, named after a semblance of the left ventricle in systole with an octopus trap, is characterized by transient, reversible, apical ballooning with preservation of basal ventricular function, commonly seen in postmenopausal women after emotional or physical stressors [1, 2]. We present a case of TTC which developed during a hospital stay for pneumonia suspected secondary to rapidly increasing platelet count in an elderly gentleman with a history of polycythemia vera (PV).

## 2. Case Description

**Figure 1:**
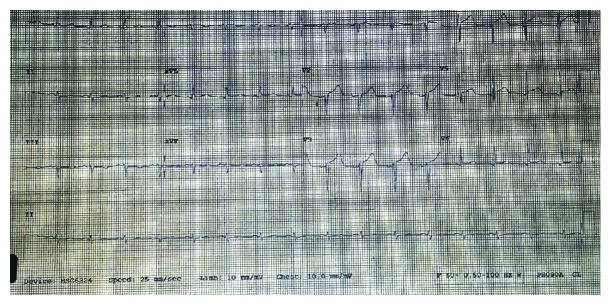
12-lead electrocardiogram showing ST-segment elevation in the anterolateral precordial leads with QT/QTc interval prolongation.

The patient is a 75-year-old man referred to our facility for the further management of ST-elevation myocardial infarction (STEMI). He had presented 3 days earlier to an outside hospital with shortness of breath and productive cough of one-day duration. The evaluation was positive for right-sided pneumonia and was started on intravenous ceftriaxone and oral azithromycin. He was gradually improving but on day 3 of admission developed worsening shortness of breath with tachypnea and oxygen desaturation. He denied any precordial pain. His electrocardiogram (EKG) showed ST-segment elevation in anterolateral precordial leads with QT/QTc interval prolongation (Figure 1). Troponin T then was 0.01 ng/mL (reference range: <0.01 ng/mL). Chest X-ray (CXR) showed diffuse pulmonary edema. He was started on intravenous (IV) furosemide and intubated for hypoxic respiratory failure. He was then transferred to our facility for expectant cardiac catheterization.

On presentation, the patient was intubated, and history was obtained from the patient's family. According to them, the patient was functionally active at baseline prior to presentation. He ambulated with a cane but was able to go up a flight of stairs without anginal symptoms. His past medical history was significant for heart failure with reduced ejection fraction, polycythemia vera, obstructive sleep apnea, hypertension, hyperlipidemia, aortic stenosis, anemia, chronic kidney disease stage IV, and stroke. He had undergone cardiac catheterization 2 years ago which had shown 40–50% stenosis of ostial left anterior descending (LAD) artery and 50% stenosis of the first diagonal branch. The patient was diagnosed with JAK2 V617F mutation-positive polycythemia vera 22 years ago and was maintained on hydroxyurea and darbepoetin alfa. Hydroxyurea was discontinued 2 weeks prior to presentation for anemia (hemoglobin 7 g/dL; reference range: 14–17 g/dL) requiring a blood transfusion and was started on ruxolitinib. Hydroxyurea was restarted 2 days prior at the referring hospital for worsening thrombocytosis.

**Figure 2:**
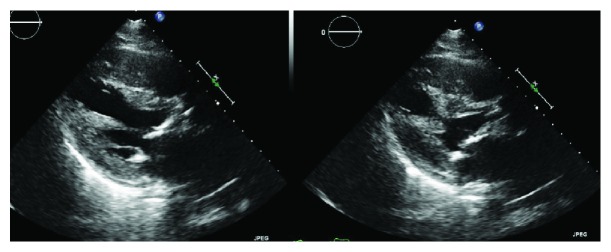
Transthoracic echocardiography with akinetic distal half of both left and right ventricles with resulting left ventricle outflow tract obstruction from the hyperkinetic basal myocardium.

**Figure 3:**
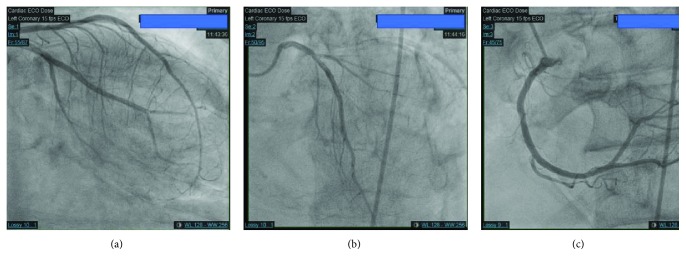
Cardiac catheterization with nonobstructive coronary artery disease of (a) left anterior descending and left circumflex arteries, (b) left main and left anterior descending, and (c) right coronary artery.

On examination, the patient was afebrile with a pulse of 80 beats per minute (bpm), blood pressure of 98/56 mmHg, respiratory rate of 28/min, and 99% saturation on FiO2 of 70% intubated on assist control mode of ventilation. Chest examination revealed diffuse crackles and aortic stenosis murmur. He did not have pedal edema. His platelet count was 3,321,000/*μ*L (reference range: 140,000–400,000/*μ*L) and was described as giant platelets on peripheral smear. During the stay, his platelet count peaked at 4,145,000/*μ*L. His white cell count was 13,100/*μ*L (reference range: 4000–11,000/*μ*L), and hemoglobin was 7.5 g/dL. Creatinine was elevated at 3.1 mg/dL (reference range: 0.7–1.4 mg/dL; baseline: 2.3–2.5 mg/dL) His probrain natriuretic peptide (pro-BNP) was 12,885 pg/mL (reference range: <450 pg/mL). Troponin T was 0.99 ng/mL. As EKG in the outside hospital showed ST elevation, he received 2 units packed red cells to reach goal hemoglobin between 9–10 g/dl. Repeat hemoglobin was 9.3 g/dL. He was continued on dual antiplatelet therapy and metoprolol tartrate 50 mg twice daily. He was started on heparin infusion. Transthoracic echocardiography showed an ejection fraction of 35% and moderately decreased left ventricular systolic function with dyskinetic to akinetic distal half of both ventricles consistent with takotsubo cardiomyopathy (Figure 2). Cardiac catheterization revealed nonobstructive coronary artery disease (Figure 3). Heparin infusion was discontinued. Post catheterization, his hospital stay was uneventful. He was successfully extubated and discharged a week later (hydroxyurea 1000 mg and ruxolitinib 5 mg both twice daily).

## 3. Discussion

Several theories have been postulated to explain the pathogenesis of TTC. The leading mechanism is direct myocardial damage from increased catecholamine release [1]. Acute stressors activate central and peripheral adrenergic sympathetic nervous system stimulating massive amounts of catecholamine secretion [1, 3]. This leads to increased cardiac workload with coronary microvascular spasm, consequent demand ischemia, and postischemic stunning [3]. This effect is profound in the apical myocardium where negatively inotropic *β*-adrenergic receptors are abundant. This differential distribution leads to the characteristic takotsubo-like appearance on echocardiography. In postmenopausal women, the loss of the effect of estrogen exaggerates the responses of central neurons and cardiac cells and possibly attenuates the production of cardioprotective substances. Abnormalities in glucose or fatty acid metabolism and excessive coronary vasoconstriction from defect in endothelial relaxation are other proposed mechanisms for the development of TTC [1].

## 4. Polycythemia Vera

TTC has previously been reported in association with esophageal carcinoma [4]. It has not been described in association with PV. Polycythemia vera (PV) is a myeloproliferative disorder with an increased risk of both arterial and venous thromboembolism including acute myocardial infarction through increased risk for clot formation [5]. In most patients with PV, elevated hemoglobin > 18.5 g/dL is associated with thrombocytosis (platelet count > 450,000/*μ*L in 53%) [6]. Contrastingly, our patient was anemic and had nonobstructive coronary artery disease on cardiac catheterization. His platelet counts were rapidly increasing prior to presentation possibly due to discontinuation of hydroxyurea. Platelets are one of the earliest responders at the site of injury. Their antimicrobial properties come from releasing cytokines and internalizing microorganisms [7]. They also have stores of adrenaline which are released at the same time [8]. We suspect an exaggerated adrenaline release from an abnormally elevated number of platelets might have acted as a stressor and triggered TTC.

## 5. Clinical Presentation

TTC is estimated to account for 1% to 2% of cases among patients presenting with acute coronary syndrome [1]. Deshmukh et al. reported that it was exceedingly common in women than in men, more so in women > 55 years of age [9]. There is usually an identifiable physical (36.0%) trigger like surgery, trauma, infection, and exacerbation of lung disease or emotional trigger (27.7%) [1, 10]. Common presenting complaints include chest pain, shortness of breath, and syncope. On rare occasions, cardiac arrest, cardiogenic shock, and life-threatening ventricular arrhythmias may occur [1]. Cardiogenic shock is commonly seen in the setting of left ventricular outflow tract (LVOT) obstruction from basal hypercontractility [11]. Our patient had demonstrated LVOT obstruction but was not in cardiogenic shock.

## 6. Diagnosis

Mayo Clinic criteria proposed for the diagnosis of TTC requires fulfillment of 4 conditions [12]:

1. Transient hypokinesis, akinesis, or dyskinesis of the left ventricular mid segments with or without apical involvement; the regional wall motion abnormalities extending beyond a single epicardial vascular distribution; a stressful trigger often but not always present
2. The absence of obstructive coronary disease or angiographic evidence of acute plaque rupture
3. New electrocardiographic abnormalities (either ST-segment elevation and/or T-wave inversion) or modest elevation in cardiac troponin
4. The absence of pheochromocytoma and myocarditis

We did not work up our patient for pheochromocytoma or myocarditis during his hospital stay. However, he had an old imaging of his abdomen which was negative for any adrenal mass. Clinical history did not co-relate with the presentation of pheochromocytoma or myocarditis. Wall motion abnormality on echocardiography with characteristic apical ballooning and basal hypercontractility makes TTC more likely. Our patient had ST-segment elevation and QT/QTc interval prolongation on EKG which is characteristic for TTC [1, 10]. Regarding cardiac biomarkers, troponin is only modestly elevated in TTC in comparison to STEMI [1] whereas brain natriuretic peptide (BNP) levels are usually higher in TTC than in STEMI [3, 10]. Although levels of both cardiac biomarkers may have been confounded by his stage IV chronic kidney disease, our patient had manifold elevation in pro-BNP than troponin T. Coronary angiography typically shows normal coronary arteries or nonobstructive coronary artery disease with wall motion abnormalities that extend beyond the distribution of a single coronary artery [1, 3, 10]. Thus, coronary atherosclerosis does not exclude a diagnosis of TTC [10].

## 7. Treatment

TTC is initially managed as acute myocardial infarction with oxygen, aspirin, intravenous heparin, and beta blockers [1, 11]. Cardiac catheterization should not be delayed with suspicion for TTC [10]. Once confirmed, aspirin can be discontinued in the absence of coronary artery disease [1, 11]. Intravenous heparin should be continued in the absence of contraindications to prevent left ventricular apical thrombus formation. This should be bridged to warfarin which should be continued until documented resolution of regional wall motion abnormalities. In the presence of an LVOT obstruction, nitroglycerin or inotropes should be avoided as it can worsen obstruction [11]. TTC usually resolves in a few days to weeks [3, 11]. The prognosis is favorable with in-hospital mortality ranging from 0 to 8% [11].

## 8. Conclusion

This is the first case to relate excess adrenaline release from platelets as a physical stressor in a patient with PV leading to TTC. We hope this case will bring forward other similar observations and help in better understanding the underlying pathogenetic mechanism.
